# Pharmacological strategies to reduce pruritus during postoperative epidural analgesia after lumbar fusion surgery - a prospective randomized trial in 150 patients

**DOI:** 10.1186/1754-9493-5-10

**Published:** 2011-05-14

**Authors:** Eva Gulle, Carola Skärvinge, Karin Runberg, Yohan Robinson, Claes Olerud

**Affiliations:** 1 Stockholm Spine Center, Upplands Väsby, Sweden; 2 Uppsala University Hospital, Institute for Surgical Sciences, Department of Orthopaedics, Uppsala, Sweden

## Abstract

**Background:**

Epidural analgesia with bupivacain, epinephrine and fentanyl provides excellent pain control after lumbar fusion surgery, but pruritus and motor block are frequent side effects. Theoretically epidural ropivacain combined with oral oxycodone could decrease the incidence of these side effects. The two regimens were compared in a prospective randomized trial.

**Patients and methods:**

150 patients (87 women) treated with posterior instrumented lumbar fusion were included. The mean age was 51 +/- 11 years. 76 were randomized to bupivacain, epinephrine and fentanyl (group B) and 74 to ropivacain and oxycodone (group R). Pruritus, motor block and pain were measured 6 hours after surgery, thereafter 6 times per day for 5 days. Any pain breakthrough episode was registered whenever it occurred.

**Results:**

The epidural treatment could be performed in 143 patients (72 in group B and 71 in group R). Disturbing pruritus occurred in 53 patients in group B compared to 12 in group R (p < 0.0001). Motor blockade was most frequent on day 1, occurring in 45% of the patients with no difference between the groups. Both regimes gave good pain control with average VAS under 40, but the pain relief was statistically better in group B. The number of pain breakthrough episodes did not differ between the groups.

**Conclusions:**

Pruritus could be reduced with a combination of epidural ropivacain and oral oxycodone, at the price of a slightly higher pain level. Ropivacaine was not found to be superior to bupivacaine with regard to motor blocks.

## Introduction

The postoperative pain level after posterior instrumented lumbar spine fusion surgery is generally high, thus an efficient pain control regime is required. Continuous epidural analgesia (CEA) with bupivacain, epinephrine and fentanyl for three days followed by oral analgesics is an effective postoperative treatment option [1,2]. This regime has been used successfully for many years, but pruritus and motor blocks are frequently encountered side effects. Pruritus is considered to be caused by epidural administration of opiates [3,4]. Unbearable pruritus leads to an early termination of CEA in multiple cases exposing the patients to an increased risk of a pain breakthrough before the alternative pain regime has reached its full effect. Furthermore, any paresis in the postoperative period after spine surgery has to be distinguished from a serious complication to the operative procedure itself. Thus, in case of a motor block the CEA has to be stopped and the patient monitored closely until motor function returns, which may lead to temporary insufficient pain control. Pain breakthrough episodes may also occur in the transition to oral medication at the end of CEA treatment before the oral treatment has reached full effectiveness.

CEA with ropivacain combined with oral slow release oxycodone has theoretical advantages to CEA with bupivacain, epinephrine and fentanyl. Avoiding epidural opioids may decrease the incidence of pruritus. Additionally, the motor block effect of ropivacain is considered to be lower than that for bupivacain. Furthermore the risk for pain breakthrough episodes at time of epidural removal is lower if the patient already is treated with an oral slow release opiate when the CEA effect wears off.

In an unpublished small non-randomized pilot study comparing CEA with bupivacain, epinephrine and fentanyl to CEA with ropivacain combined with intravenous opiates, we saw a decrease in pruritus and motor blocks and fewer problems with pain breakthrough episodes during treatment and at discontinuation when ropivacain was used. Therefore a randomized trial would be of value to investigate whether the postoperative period could be significantly improved for lumbar spine fusion patients, using a different epidural analgesia protocol.

The aim of this study was to compare the effectiveness of two different pain control regimes after posterior instrumented lumbar fusion surgery for degenerative conditions in a randomized trial.

### Patients and methods

The study was approved by the Regional Ethics Committee in Stockholm (2007-001417-41), and written informed consent was obtained from all subjects.

Included were patients between 18 and 70 years of age, being treated with one to four level posterior instrumented lumbar spine fusion for degenerative conditions. Exclusion criteria were: use morphine analgesics before surgery, known allergy for any of the used drugs, any known contraindication to CEA, psychiatric disease or language problems, which would have led to great difficulties understanding the instructions or communicating the results.

The series consisted of 150 patients (87 women and 63 men), with a mean age of 51 ± 11 years. Seventy-six were randomized to group B and 74 to group R after having received written information and given consent. The baseline demographics, indications for surgery, and preformed procedures are listed in Table 1. There were no differences between the groups with regard to baseline data.

**Table 1:** Baseline data of the two treatment groups.

		Group B	Group R
Women/men		43/33	44/30

Age ± SD		51.6 ± 10.2	50.2 ± 10.6

Diagnosis	Spondylolysis/-olisthesis	17	13
	
	Disk herniation	2	4
	
	Spinal stenosis	26	18
	
	Degenerative disc disease	28	34
	
	Non-union of previous fusion	3	5

Treatment	Decompression and posterior fusion	27	24
	
	Posterior fusion without decompression	16	21
	
	Posterior lumbar interbody fusion	33	29

Levels fused	1	53	43
	
	2	19	27
	
	3	4	2
	
	4	0	2

### Treatment protocol

One hour before the operation the premedication was given. The patients were operated under general anaesthesia. In both treatment groups an 18 G epidural catheter was inserted openly with the tip at the lower thoracic region towards the end of the operation. The CEA was continued for three days. The patients in both groups adjuvant paracetamol 1 g × 4 was included in the treatment. For the detailed CEA protocols see Table 2.

**Table 2:** CEA protocols for group B (standard protocol) and group R (experimental protocol)

	Group B	Group R
premedication	1 g oral paracetamol.	20 mg oral slow release oxycodone and 1 g oral paracetamol.

CEA/analgesia solution	Bupivacain 1 mg/ml, Epinephrine 2 μg/ml, and Fentanyl 2 μg/ml.	Ropivacain 2 mg/ml, combined with 20 mg oral slow release oxycodone with 12 hours interval.

Test dose before wound closure	2 ml bupivacain 5 mg/ml with epinephrine 5 μg/ml.	2 ml ropivacain 5 mg/ml with epinephrine 5 μg/ml.
	
	If no adverse reaction was seen during a few minutes another dose of 5 to 7 ml was given.	

CEA dosage	Initial dose 4 to 6 ml per hour combined with patient administered bolus doses of 2 ml with a maximum of 3 boluses per hour.	
	
		The slow release oxycodone was continued twice a day.

Duration of CEA	72 hours, transition to oral slow release oxycodone 20 mg twice a day.	72 hours, while the oral slow release oxycodone treatment was continued.

Pain breakthrough rescue treatment	Boluses of 2-6 ml were given and/or the epidural catheter was manipulated if indicated.	
	
	If the initial treatment would fail CEA was exchanged to bupivacain-only combined with patient controlled intravenous opiate, morphine or ketobemidone.	If this initial treatment would fail extra oral oxycodone or intravenous opiates, morphine or ketobemidone, may be added. If also this would fail patient controlled intravenous opiates was applied.

### Sample size calculation

Pruritus was the primary variable of interest. In our pilot study pruritus was present in 40% of patients receiving CEA with bupivacain, epinephrine and fentanyl. A decrease to 10% would be considered a clinically successful reduction. With 5% significance level and 80% power 49 patients would have to be included in each group. Corresponding figures for motor blockade is 35 patients and for pain breakthrough episodes 67 patients. As CEA presumably must be terminated in several cases because of a lack of effect we chose to include 75 patients in each group to cover for these and other losses.

### Randomization

Randomization was done using numbered closed envelops which were filled in accordance to a computer generated randomization list. The patients were given envelopes consecutively as they were entered into the study. The envelope was opened and the patient informed when the premedication was given just before the patient was brought to the OR.

### Data collection

Pruritus was measured according to an arbitrary scale: 0 - no pruritus; 1 - mild pruritus not requiring any medical treatment; 2 - moderate pruritus requiring medical treatment; 3 - severe pruritus leading to discontinuation of the treatment. The highest score for each day was used for the further analysis.

Motor blockade was registered according to Bromage [5]: 0 - Free movements of legs and feet; 1 - Just able to flex knees, free movements of feet; 2 - Unable to flex knees, free movements of feet; 3 - Unable to move legs or feet. The highest score on the worst side for each day was used for the further analysis.

Pain was measured with a visual analogue scale (VAS) from 0 (no pain) to 100 (worst possible pain) [6]. A mean VAS score for each day was calculated and used for the further analysis.

All patients were evaluated on a set time schedule every fourth hour for five days. However, if the patient was sound asleep during the night the analgesia effect was considered satisfactory and he/she was not awakened for data acquisition. In addition an extra registration was done at six hours after surgery.

A pain breakthrough episode was defined as VAS ≥70 and the number of these events was registered for each patient, as was the number of extra doses of opiates given.

### Statistical Analysis

The primary variable pruritus was dichotomised as absent, i.e. score 0 or 1, or present, i.e. score 2 or 3, registered at any time during the study period. The two study groups were then compared using Fisher's exact test. Presence of pruritus was also compared between the treatment groups with repeated measurement ANOVA, as was presence of motor blockade and pain scores. The number of pain breakthrough episodes and extra doses of opiates were compared with non-parametric tests. P < 0.05 was considered significant.

## Results

One-hundred-and-fifty patients were included in the study; 76 were randomized to group B and 74 to group R. The CEA treatment could not be initiated in 7 patients: one patient in group B developed a stroke and died shortly after the operation; a further investigation ruled out any association to the applied pain treatment. The epidural catheter was not functional in 6 cases most likely due to occlusion or dislocation.

**Figure 1:**
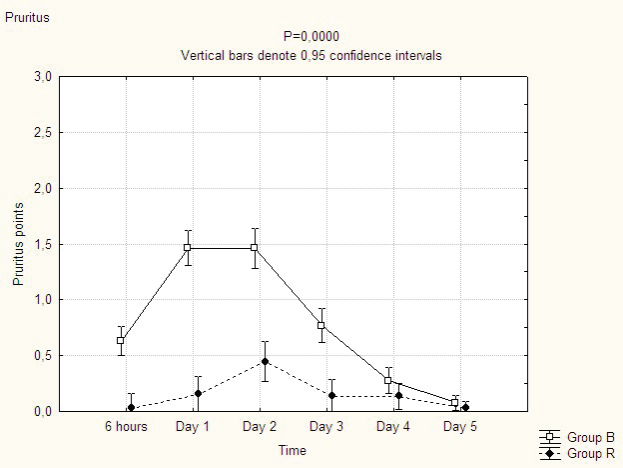
**The development of pruritus over time**.

Clinically relevant pruritus (grade 2 or 3 according to our scale) was present in 74% in group B compared to 17% in group R, P < 0.0001 (Table 3). In group B the elevated pruritus level was present already at 6 hours after the operation, reached a maximum at day 1 and 2, and remained elevated until the CEA was removed at day 4 whereas the pruritus level in group R was fairly constant during the entire study period (Figure 1).

**Table 3:** Summary of daily pruritus level counts.

	Group B					Group R				
day	1	2	3	4	5	1	2	3	4	5
**Pruritus**										
**0**	12	10	29	50	62	59	46	61	61	66
**1**	11	17	24	14	3	6	12	5	5	2
**2**	43	39	13	2	1	3	10	2	2	0
**3**	0	0	0	0	0	0	0	0	0	0

The number of patients with clinical relevant pruritus, i.e. defined as grade 2 or 3 in our scale, in the two treatment groups differed significantly in favour of group R, P < 0.0001.

**Figure 2:**
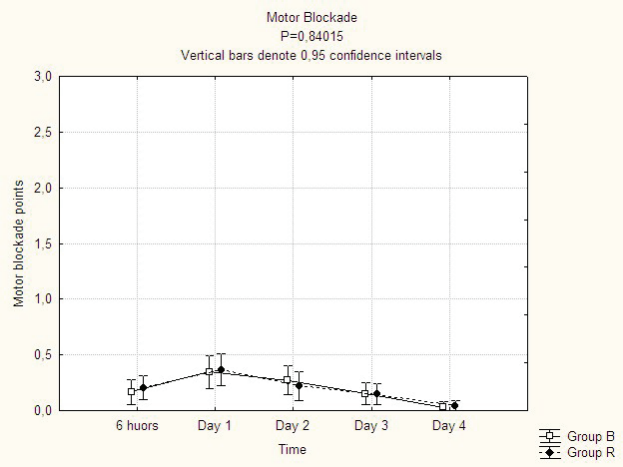
**The development of motor blockade over time**.

Motor blockade varied over time reaching a maximum during day 1 when 45% of the patients were affected. However, the motor blockade was in the majority of cases of the mildest form, and more pronounced problems were seldom seen. There was no difference between the treatment groups (Figure 2 and Table 4).

**Table 4:** Motor blockade according to Bromage [5]

Motor blockade level	Group B					Group R				
	6 h	Day1	Day2	Day3	Day4	6 h	Day1	Day2	Day3	Day4
0	59	48	51	57	64	59	50	58	59	65
1	11	17	13	8	2	10	18	10	8	3
2	1	6	3	1	0	2	2	1	1	0
3	0	0	0	0	0	0	1	1	0	0

**Figure 3:**
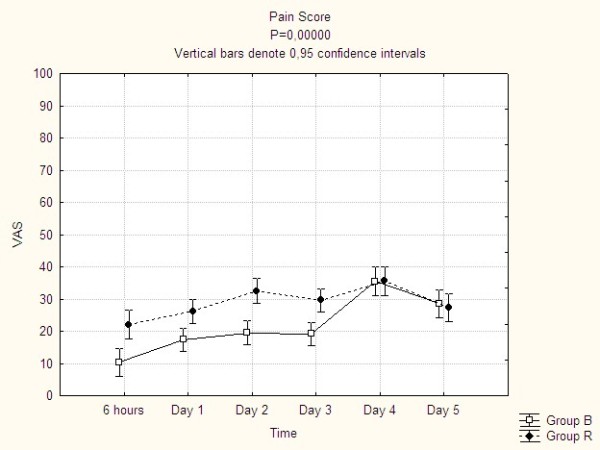
**The development of pain over time**.

Pain control was very effective in both treatment groups with the mean VAS-value regularly below 40. There was, however, a statistically significant difference between the groups in favour of group B throughout the CEA treatment period of 10-14 VAS units. There was also a statistically significant increase in VAS-value in group B during the day of CEA removal (Figure 3). There was no difference between the treatment groups with respect to pain breakthrough episodes: 83 of the patients experienced 303 pain breakthrough episodes during the entire study period, 40 patients and 126 episodes in group B compared to 43 patients and 177 episodes in group R (n.s.). Pain breakthrough episodes during day 4, i.e. the day of CEA removal, amounted to 25 patients in group B compared to 17 patients in group R (n.s.). 76% of the patients in both treatment groups received extra doses of opiates during day 4 without any significant difference between the groups (Table 5).

**Table 5:** The total number of patients with pain breakthrough episodes during the study period and during day 4,  i.e. the day of CEA removal, and the number of patients receiving extra doses of opiates during day 4.

	Group B	Group R	P value
Patients with pain breakthrough episodes total/n	40/66	43/69	0.49

Patients with pain breakthrough episodes day 4/n	25/66	17/68	0.08

Patients receiving extra doses of opiates day 4/n	54/66	55/68	0.89

In group B 5/72 patients and in group R 3/71 had to discontinue the CEA treatment prematurely (n.s.). In group B: 3 patients due to severe pruritus and 2 patients due to unsatisfactory pain control. In group R: 2 patients due to motor blockade and 1 patient due to unsatisfactory pain control. In addition 3 patients, all in group R, were discharged home already on day 4 because they were fully mobilized and no longer in need of hospital care. No data were collected on day five for these patients. The parameters for all these patients were included in the group analysis for the time they were available for the study.

There were no gender differences.

## Discussion

In the presented randomized trial dramatically lower pruritus levels were found if epidural use of opioids was avoided. This confirms the findings in the recently published randomized trial of Prasartritha et al [7] who found increased pruritus if epidural fentanyl was used after lumbar spine surgery. Postoperative pain levels as measured with VAS were lower in our study if opoiods were administered epidurally. Thus the investigated experimental CEA protocol using ropivacain and oral opioids seems to be less effective with regard to pain control.

Apart from good pain control the application of CEA is advantageous in multiple regards: First the lung function and blood flow in the legs are improved. Then the neuro-endocrine stress reaction and the myocardial oxygen consumption are reduced and intestinal motility is stimulated. This leads indirectly to a reduction in pulmonary complications, thromboembolic episodes, as well as other complications, shortens the hospital stay, and decreases hospital costs [8]. The best pain control seems to be if a local anaesthetic is combined with opiates in the CEA and the combination of bupivacain, epinephrine and fentanyl, has been recommended [1,9]. However, we have experienced frequent side effects with pruritus and motor blockades as the most common ones. Other studies reports side effects as pruritus related to epidural opioids [3,4]. In a study of 1014 patients treated with CEA after major general surgical procedure 82.6% had a good or excellent pain relief. They had used a mixture of bupivacain and fentanyl and just as in our study they encountered a high incidence of pruritus. Other side effects related to fentanyl were sedation and nausea, and to bupivacain were sensory disturbances and motor blockades in the legs [2].

To avoid the side effects an ideal postoperative pain regime would combine CEA using a drug with less motor blockade effect than bupivacain with an opiate administered in a way not leading to pruritus, i.e. not epidurally. Ropivacaine has a milder side effect profile with less risk of motor blockades but with equal pain control potential as bupivacain [10], which makes this an interesting drug to investigate. Opiates could be administered both orally and intravenously. On the one hand intravenous opioid infusion would be easy to control but would require an additional infusion pump which is awkward and resource consuming, and the transition to oral analgesics on day four would still be a potential problem. On the other hand if oral slow release administration could be used already from the start the problem of transition to oral medication would be eliminated. Oral slow release oxycodone has been shown to be an effective agent in postoperative pain treatment in abdominal surgery, where it, in combination with infiltration of the wound with 25-40 ml 0.25% bupivacaine was equipotent to CEA with bupivacain [11]. The combination of CEA using ropivacain and oral slow release oxycodone was chosen for the present study. This would combine the positive effects of CEA with improved pain control of a systemic opiate. Another benefit would be that the patient could continue with the same oral analgesics after the CEA treatment, which possibly could lower the risk of pain breakthrough episodes. The present study was conducted in order to compare this combination with the traditional CEA with bupivacain, epinephrine and fentanyl.

Being the most common side effect, leading regularly to an early termination of CEA, pruritus was chosen as our primary variable. We could demonstrate that pruritus could be decreased to a great extent, but not be eliminated when avoiding epidural morphine. Therefore it is likely that postoperative pruritus is influenced by further variables. For instance lying in bed for many hours with wound dressings taped to the skin, in unfamiliar bed sheets washed with unfamiliar detergents may still contribute to itching sensations even after the pharmacological effect of epidural opiates have been removed.

In our previous pilot study we saw motor blockades quite frequently in CEA with bupivacain, epinephrine and fentanyl (unpublished data). Because of its different pharmacological properties we hypothesized that this complication would have been less frequent when using ropivacain. However motor blockade was reported by almost half of the patients irrespective of treatment. Thus, it seems like this complication is a rather unavoidable part of CEA. Beyond that, the presented data could not confirm a lesser effect on motor function of ropivacain compared to other local anaesthetics, as claimed by the manufacturer. Attempts to decrease this effect by reducing the dose of ropivacain will most likely also decrease the pain controlling effect.

The pain level was significantly higher in the new treatment compared to the traditional one, which is still an unaddressed problem of this oral opioid medication. However, the pain levels in both groups were generally low, with the mean value well below 40 VAS-units. The increased pain level in group R never constituted a clinical problem. The difference between the groups were in the range of 10-14 VAS-units with is hardly of clinical relevance. We have not found any reference values for the minimally clinically important difference (MCID) after surgery, but for low back pain patients it has been estimated to be 18 of 100 VAS-units [12]. The small difference in pain control between the treatments is also supported by the fact that the total number of pain breakthrough episodes did not differ between the groups. On the contrary, the group with bupivacain, epinephrine and fentanyl had a tendency towards more pain breakthrough episodes during the day of CEA removal.

## Conclusion

The pain control regime consisting of CEA with ropivacain combined with oral slow release oxycodone reduced the problem with pruritus when compared to CEA with bupivacain, epinephrine and fentanyl, probably due to the elimination of epidural opiates. The reduction in pruritus was associated with an increase in pain score. Low grade motor blockade was equally frequent during CEA treatment in both groups even though more severe grades seldom occurred. The claim by the manufacturer that ropivacain results in less effect on motor function than other local anaesthetics could not be confirmed.

## Competing interests

The authors declare that they have no competing interests.

## Authors' contributions

EG, CS, KR, and CO were clinical investigators of this study. EG, CS, and CO analysed the data and wrote the manuscript, and CO and YR critically revised it. All authors read and approved the final manuscript.
